# Cutaneous Manifestations of Thyroid Disease: A Case of Thyroid-Induced Myxedema

**DOI:** 10.1155/2011/386081

**Published:** 2011-11-30

**Authors:** Anjali Shroff, Gregory Simpson

**Affiliations:** ^1^Medical College of Georgia, Augusta, GA 30004, USA; ^2^Department of Dermatology, University of California, Fresno, CA 93701, USA; ^3^University Dermatology Associates, 2335 E. Kashian Lane, Suite 410, Fresno, CA 93701, USA

## Abstract

The cutaneous manifestations of thyroid disease can be present in the hair, nails, and local or diffuse locations throughout the skin. Traditionally, thyroid-associated mucin deposition is present in a pretibial location on bilateral lower legs. We present a case of a growing plaque on the lower back of a 10-year-old girl, whose appearance coincided with a recent diagnosis of Hashimoto's thyroiditis.

## 1. Introduction

A 10-year-old Hispanic girl presented with a two-year history of an asymptomatic, growing plaque on the lower mid-back. She had tried ammonium lactate 12% lotion topically for 3 months without any success. She was diagnosed with Hashimoto's thyroiditis approximately 3 years prior, after having a swollen thyroid gland on physical exam. After 2-year treatment with levothyroxine she is presently controlled without any systemic medication, and her only physical manifestation is a tendency to be cold. She has had no other cutaneous issues such as hair loss or nail problems but did report an increase in overall dryness of skin. Her only medication at the time of exam was occasional Montelukast for seasonal allergies.

**Figure 1:**
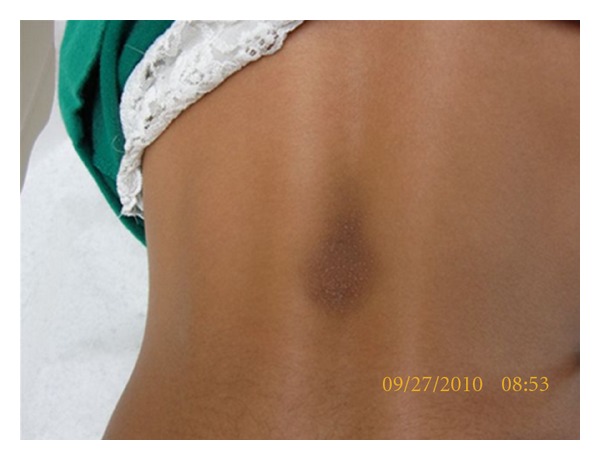
Hyperpigmented plaque on midback.

**Figure 2:**
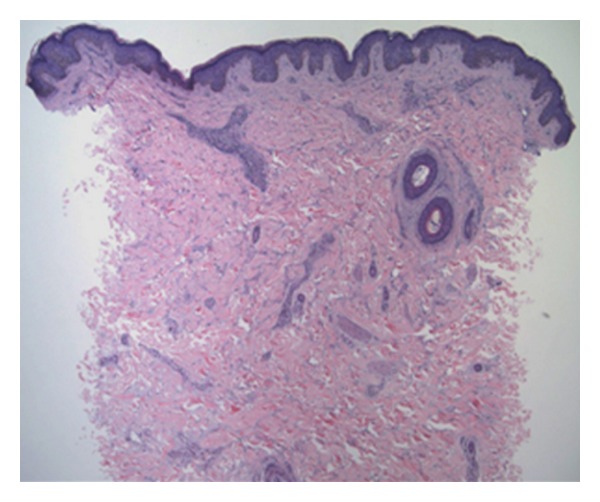
Low power (4X) sparse perivascular infiltrate.

**Figure 3:**
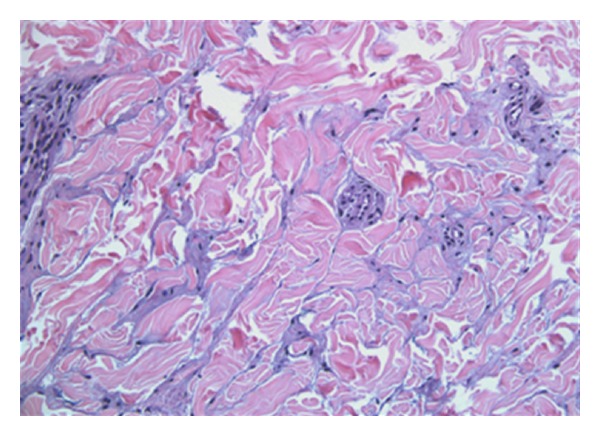
High power (40X) increase in dermal mucin.

On examination, the child was alert and oriented, with no evidence of any developmental abnormalities. There was a soft, hyperpigmented 4.5 × 2.0 cm plaque on the midback region with a slight peau d'orange texture (Figure 1), while the rest of her body had no unusual cutaneous findings. Histopathology by H&E stain showed an increased amount of connective tissue mucin (Figure 2) in the dermis associated with a sparse perivascular infiltrate (Figure 3).

## 2. Discussion

Both hypo- and hyperthyroidism are highly associated with various cutaneous changes. Hypothyroidism is generally associated with thick, dry skin, livedo reticularis of the extremities and thinning of dry, brittle hair with extent of disease dictating severity of manifestations [1]. These effects are due to a decreased metabolic rate which causes peripheral vasoconstriction and decreased sebaceous gland secretion [2]. Hyperthyroidism is associated with thin, soft hair with possible progression to alopecia, onychodystrophy, and hyperpigmentation of the hands, feet, and mouth [2]. Hashimoto's thyroiditis usually has clinical manifestations similar to hypothyroidism, but it has an autoimmune etiology similar to Graves' disease, a common cause of hyperthyroidism. Due to this shared etiology in both diseases, similar dermatologic and ophthalmologic findings are seen such as conjunctivitis, diplopia, and blurred vision along with a cutaneous myxedema that can occur in pretibial, scalp, or preradial distributions [1]. In other respects, Hashimoto's cutaneous manifestations are very similar to hypothyroidism such as thick scaly skin and brittle hair.

Graves' disease is most commonly associated with ophthalmopathy, pretibial myxedema, and moist, pruritic skin [1]. Pretibial myxedema can occur in other distributions such as arms, shoulders, neck, and upper back [2]. Other manifestations include hyperpigmentation, yellowing, and possibly disfiguration of the nails [2].

In this 10-year-old girl with a history of Hashimoto's thyroiditis, the plaque appeared shortly after diagnosis and grew slowly over the next 2 years. Biopsy revealed an increase in dermal mucin which could be secondary to myxedema (generalized versus pretibial versus lichen), collagen vascular diseases (lupus erythematosus, dermatomyositis), or focal cutaneous mucinosis. She had a negative connective tissue disease workup (ANA, DNA antibodies), and clinically the lesion was a localized, raised, waxy plaque reminiscent of classic pretibial myxedema. The patient and mother described a waxing and waning course of Hashimoto's over the last two years; however laboratory evaluation of the disease is minimal.

In conclusion, we present a young girl with history of thyroid disease and a hyperpigmented, growing plaque suspicious for thyroid-induced myxedema to her lower back. When assessing patients with thyroid disease, it is important to perform a detailed cutaneous examination, as the manifestations of disease are extremely variable.

## References

[1] Burman KD, McKinley-Grant L (2006). Dermatologic aspects of thyroid disease. *Clinics in Dermatology*.

[2] Doshi DN, Blyumin ML, Kimball AB (2008). Cutaneous manifestations of thyroid disease. *Clinics in Dermatology*.

